# Acute Kidney Injury in Patients With Suspected Pulmonary Embolism: A Retrospective Study of the Incidence, Risk Factors, and Outcomes in a Tertiary Care Hospital in Saudi Arabia

**DOI:** 10.7759/cureus.21198

**Published:** 2022-01-13

**Authors:** Abdulrahman M Alhassan, Ahmad Aldayel, Abdullah Alharbi, Mahfooz Farooqui, Mohammed H Alhelal, Faisal Alhusain, Abdulkareem Abdullah, Mohammed Altoyan

**Affiliations:** 1 Medicine, King Abdulaziz Medical City, National Guard Health Affairs, Riyadh, SAU; 2 Nephrology, King Abdulaziz Medical City, National Guard Health Affairs, Riyadh, SAU; 3 Emergency Medicine, King Abdulaziz Medical City, National Guard Health Affairs, Riyadh, SAU

**Keywords:** pe, aki, contrast-induced nephropathy, pulmonary embolism, acute kidney injury

## Abstract

Background

Pulmonary embolism (PE) is a known cause of morbidity and mortality. A diagnosis of PE is made by computed tomography pulmonary angiogram (CTPA) or a ventilation-perfusion (V/Q) scan. This study aimed to assess the incidence and predictors of acute kidney injury (AKI) in patients with suspected PE.

Methods

This study was a retrospective study including patients with suspected PE who underwent a CTPA and/or a V/Q scan from 2015 to 2020. The patients were grouped into CTPA or V/Q scan. Creatinine levels were obtained before and after the procedure. AKI was defined based on an increased serum creatinine by 0.3 mg/dL within 48 hours.

Results

A total of 752 patients were included in the study. The majority (n = 688) underwent a CTPA as a diagnostic modality in patients suspected to have pulmonary embolism (PE), and a V/Q scan was used in 73 patients. Of the 752 patients, there were eight patients who underwent both diagnostic modalities. PE was diagnosed in 121 (16.1%) patients. The incidence of AKI was observed in 15.8%. PE was suspected more frequently in the female group (n = 481, 64%), with a 50% reduction of AKI risk, compared with the male group (p-value = 0.004, OR = 0.522, 95% CI = 0.337-0.81). The presence of diabetes mellitus (DM) and hypertension (HTN) was associated with AKI (p-value < 0.001). Of the AKI group, 43 (36.1%) patients had malignancy. The presence of malignancy was a predictor of increased AKI risk (p-value = 0.014, OR = 1.74, 95% CI = 1.21-2.70). A small proportion (2.1%, n = 16) required dialysis. Patients who developed AKI had a 30-day mortality of 20.2% compared with 5.1% for the group without AKI.

Conclusion

In our sample, clinicians suspected PE more frequently in the female group. The overall incidence rate of AKI in patients suspected of having PE was 16.1%. The presence of diabetes mellitus and hypertension was associated with AKI. However, DM and HTN were not predictors of AKI. The risk of AKI requiring dialysis was relatively low (2.1%). There was no relationship between the diagnostic modalities and PE, and AKI, suggesting that clinicians overestimate the fear of contrast-induced AKI (CI-AKI).

## Introduction

Acute kidney injury (AKI) is defined as an abrupt deterioration of the renal parenchymal function that can be reversible over a period of days or weeks [1]. The damage is severe enough to accumulate waste products in the blood, such as urea. In addition, it results in a reduction in urine output to less than 400 mL/day in adult patients [1]. The causes of AKI are classified into three categories, depending on the cause of the injury: prerenal, intrarenal, and post-renal [2]. Approximately 130-140 persons per million population are annually diagnosed with AKI [1], and 10%-15% of hospitalized patients and 50% or more of critically sick patients develop signs of AKI [3]. In the southern province of Saudi Arabia, a study conducted over two years with 150 cases of AKI reported that 38% acquired AKI before hospital admission, and 62% developed the injury during hospital admission [4].

Pulmonary embolism (PE) is an emergency condition in which a fragment of a thrombus travels through the venous system to the lungs via the right ventricle. PE usually originates from a DVT that develops in favorable conditions known as Virchow’s triad, which indicates a hypercoagulable state, decreased endothelial integrity, and blood flow stasis. A clinical and pretest probability assessment is done first in hemodynamically stable patients with suspected PE. However, several imaging studies have been developed to facilitate this process due to the difficulty in diagnosing PE. Computed tomography pulmonary angiogram (CTPA) is the imaging modality of choice due to the high sensitivity and specificity for the workup of patients with suspected acute PE. A disadvantage of CTPA is using an intravenous contrast medium, which is considered associated with contrast-induced nephropathy. Another modality used is ventilation-perfusion (V/Q) scanning, which is an alternative to CTPA in patients with renal insufficiency and/or contrast allergy [5].

Since PE is an emergency condition, it requires a rapid diagnosis and management. The term contrast-induced acute kidney injury (CI-AKI) has been a dilemma for physicians. Some overestimate the risk and thus deprive the patients of an accurate diagnosis. On the other hand, if the risk is underestimated, it may result in a nephrotoxic insult and poor outcome. This raises the question “are patients who refuse a CTPA or choose a V/Q scan safe from AKI and its complications?” This study aimed to identify the incidence rate of AKI in patients suspected of having PE and understand its associated factors.

## Materials and methods

This was a retrospective cohort study with a sample of 752 patients with suspected PE who had a CTPA, V/Q scan, or both from January 2015 to June 2020 at King Abdulaziz Medical City, National Guard Health Affairs (NGHA) in Riyadh, Saudi Arabia. The NGHA is a tertiary center with a total bed capacity of 1973, 104 intensive care unit (ICU) beds with more than 4000 admissions per year, and an emergency department (ED) with 58 beds. In total, 7745 patients underwent imaging, and a sample of 752 was selected randomly. The sample size was calculated using Raosoft (Raosoft Inc., Seattle, WA, USA). The recommended sample size with a 5% margin of error and 95% confidence interval was 367. The inclusion criteria were adults (≥18 years) who underwent CTPA or V/Q scan to confirm the diagnosis. The exclusion criteria were patients on dialysis or without creatinine levels before or after radiological investigation.

Data were gathered through the BESTCare system from the electronic medical records. The research team reviewed the charts to confirm the imaging modalities and the diagnosis of PE and then divided the patients into CTPA or V/Q scan. Data included gender, age, BMI, presence of comorbidities, pregnancy status, history of recent surgery defined as a major operation within one month, mobility status, recent travel, Glasgow Coma Scale (GCS) score at the time of suspected PE, dialysis/renal replacement therapy within one month after the radiological imaging, and mortality within one month of suspecting the PE. Creatinine levels were obtained before and after the procedure. Based on the Kidney Disease: Improving Global Outcomes (KDIGO) criteria [6], AKI was defined based on an increased serum creatinine by 0.3 mg/dL within 48 hours from the baseline.

The Statistical Package for the Social Sciences version 26 (IBM Corp., Armonk, NY, USA) was used for the analysis. Frequency and percentage were generated for the categorical variables, and the distributed data were reported as mean and standard deviation (SD). The groups were compared with a chi-square test and considered statistically significant if the p-value was <0.05. A multivariate logistic regression model was used with a 95% confidence interval to assess AKI predictors and mortality.

## Results

In total, 752 patients were included in the study. The majority were female (64%), and 354 (47.1%) patients were ≥60 years old, with a mean age of 56.8 ± 19.5 years (Table 1). The comorbidities identified were diabetes mellitus (DM) in 321 (42.7%) patients, hypertension (HTN) in 359 (47.7%) patients, a history of malignancy in 206 (27.4%) patients, and a history of stroke in 85 (11.3%) patients. The majority (n = 688) underwent a CTPA as a diagnostic modality in patients suspected to have pulmonary embolism (PE), and a V/Q scan was used in 73 (9.7%) patients. Eight patients underwent both diagnostic modalities. The diagnosis of pulmonary embolism (PE) was confirmed in 121 (16.1%) patients (Table 1). When PE was suspected, 35 (4.7%) of the sample had a GCS score of ≤8. AKI occurred in 119 patients (15.8%), and 16 (2.1%) required urgent dialysis within one month due to acute kidney injury. The 30-day mortality in the patients with suspected PE was 7.4% (n = 56).

**Table 1:** Basic demographic data of the sample

Variables	Number (%), mean ± SD
Age	56.8 ± 19.5
≥60 years	354 (47.1%)
<60 years	398 (52.9%)
Gender	
Female	481 (64%)
Male	271 (36%)
BMI	29.4 ± 8.9
≥30	346 (46%)
<30	406 (54%)
DM	321 (42.7%)
HTN	359 (47.7%)
History of stroke	85 (11.3%)
Mobility status	
Fully mobile	497 (66.1%)
Restricted to chair	53 (7%)
Restricted to bed	202 (26.9%)
History of malignancy	206 (27.4%)
History of recent surgery	
No	621 (82.6%)
Yes	131 (17.4%)
History of travel	22 (2.9%)
Pregnancy status	
No	725 (96.4%)
Yes	6 (0.8%)
Within one month postpartum	21 (2.8%)
PE	
Negative	631 (83.9%)
Positive	121 (16.1%)
CTPA	
Not done	65 (8.6%)
Done, PE confirmed	110 (14.6%)
Done, PE ruled out	577 (76.7%)
V/Q scan	
Not done	679 (90.3%)
Done, PE confirmed	12 (1.6%)
Done, PE ruled out	61 (8.1%)
Dialysis within 30 days	16 (2.1%)
Mortality within 30 days	56 (7.4%)
AKI	
Yes	119 (15.8%)
No	633 (84.2%)
GCS score	
≤8	35 (4.7%)
>8	717 (95.3%)

**Figure 1:**
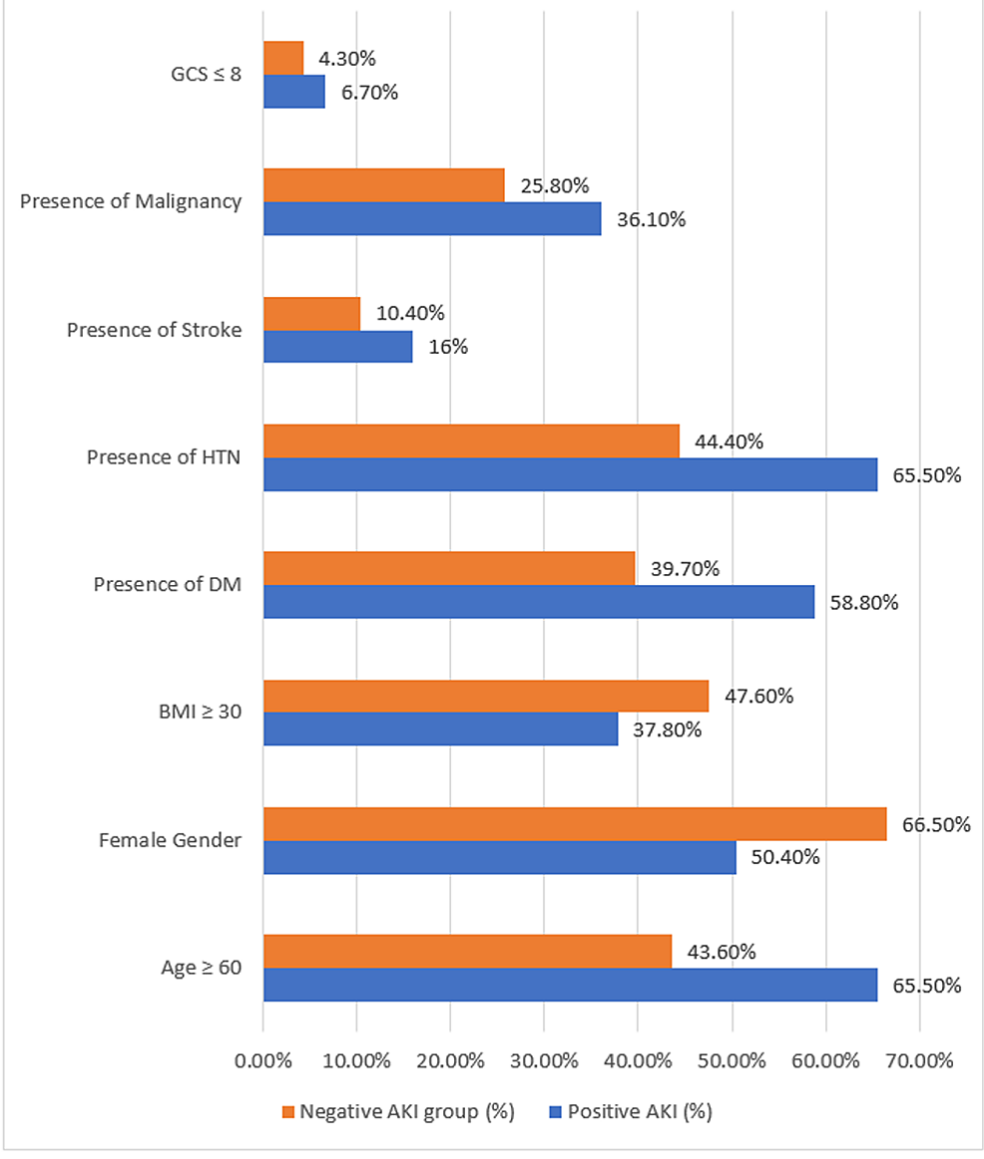
Variables in percentage in relation to AKI

The association between age and AKI was statistically significant (p-value < 0.001), with the incidence of AKI higher in the ≥60-year age group (Table 2). The AKI incidence in relation to gender was statistically significant (p-value = 0.001), and 49.6% of patients with AKI were males. The majority of the AKI group had DM and HTN (Figure 1), and there was a statistical significance between the two comorbidities and AKI (p-value < 0.001) (Table 2). More than a third (n = 43, 36.1%) of the patients with AKI had a history of malignancy. Of the 119 patients with AKI, 16 (13.4%) patients were diagnosed with PE; 14 (11.8%) patients were diagnosed using CTPA and two (1.7%) patients were diagnosed using a V/Q scan. There was no statistical significance between PE and the two diagnostic modalities (Table 2). Concerning the pregnancy status of the patients and the AKI incidence, only one patient was diagnosed with AKI in the postpartum period. Nineteen (16%) patients with AKI had a history of recent surgery, and none of the AKI group had a history of recent travel. The mortality rate in the AKI group was 20.2%, which was statistically significant (p-value < 0.001).

**Table 2:** AKI in relation to basic demographic data

Variables		AKI		P-value
		Yes (number (%))	No (number (%))	
Age	≥60 years	78 (65.5%)	276 (43.6%)	<0.001
	<60 years	41 (34.5%)	357 (56.4 %)	
Gender	Female	60 (50.4%)	421 (66.5%)	0.001
	Male	59 (49.6%)	212 (33.5%)	
BMI	≥30	45 (37.8%)	301 (47.6%)	0.051
	<30	74 (62.2%)	332 (52.4%)	
DM	No	49 (41.2%)	382 (60.3%)	<0.001
	Yes	70 (58.8%)	251 (39.7%)	
HTN	No	41 (34.5%)	352 (55.6%)	<0.001
	Yes	78 (65.5%)	281 (44.4%)	
History of stroke	No	100 (84%)	567 (89.6%)	0.08
	Yes	19 (16%)	66 (10.4%)	
Mobility status	Fully mobile	68 (57.1%)	429 (67.8%)	0.078
	Restricted to chair	10 (8.4%)	43 (6.8%)	
	Restricted to bed	41 (34.5%)	161 (25.4%)	
History of malignancy	No	76 (63.9%)	470 (74.2%)	0.20
	Yes	43 (36.1%)	163 (25.8%)	
History of recent surgery	No	100 (84%)	521 (82.3%)	0.649
	Yes	19 (16%)	112 (17.7%)	
History of travel	No	119 (100%)	611 (96.5%)	0.039
	Yes	0 (0.0%)	22 (3.5%)	
Pregnancy status	Not pregnant	118 (99.2%)	607 (95.9%)	0.205
	Yes, Pregnant	0 (0.0%)	6 (0.9%)	
	Within one month postpartum	1 (0.8%)	20 (3.2%)	
PE	Negative	103 (86.6%)	528 (83.4%)	0.39
	Positive	16 (13.4%)	105 (16.6%)	
CTPA	Not done	14 (11.8%)	51 (8.1%)	0.303
	Done, Positive PE	14 (11.8%)	96 (15.2%)	
	Done, Negative PE	91 (76.5%)	486 (76.8%)	
V\Q scan	Not done	104 (87.4%)	575 (90.8%)	0.46
	Done, Positive PE	2 (1.7%)	10 (1.6%)	
	Done, Negative PE	13 (10.9%)	48 (7.6%)	
Mortality within 30 days	No	95 (79.8%)	601 (94.9%)	<0.001
	Yes	24 (20.2%)	32 (5.1%)	
GCS	≤8	8 (6.7%)	27 (4.3%)	0.24
	>8	111 (93.3%)	606 (95.7%)	

Table 3 shows patient characteristics and comorbidities as predictors of AKI. Despite the varying incidence of AKI per age group, age was not a predictor of AKI (Table 3). A history of malignancy was a statistically significant predictor of AKI and increased the risk, compared with patients without a history of malignancy (p-value = 0.014, OR = 1.74, 95% CI = 1.21-2.70). Gender was also a statistically significant predictive variable. The risk of AKI was less in the female group by 48% (p-value = 0.004, OR = 0.522, 95% CI = 0.337-0.81). The comorbidities were not significant predictors of AKI (DM: p-value = 0.208, OR = 1.387, 95% CI = 0.834-2.307; a history of stroke: p-value = 0.650, OR = 1.158, 95% CI = 0.614-2.185). However, hypertension showed a trend toward increasing the risk of AKI (OR = 1.72, 95% CI = 0.993-2.992) but did not reach a statistical significance (p-value = 0.053). A history of recent surgery, mobility status, GCS, and BMI were not predictors of AKI (Table 3).

**Table 3:** Multivariate logistic regression of basic characteristics and comorbidities to AKI

Variables	P-value	OR	95% CI	
			Lower	Upper
Age ≥ 60 years	0.097	1.523	0.926	2.504
Gender (female)	0.004	0.522	0.337	0.810
BMI ≥ 30	0.303	0.788	0.501	1.240
DM	0.208	1.387	0.834	2.307
HTN	0.053	1.724	0.993	2.992
Stroke	0.650	1.158	0.614	2.185
Mobility status (on wheelchair)	0.580	1.240	0.578	2.659
Mobility status (restricted to bed)	0.717	1.098	0.663	1.817
Mobility status (fully mobile)	1.0			
Malignancy	0.014	1.741	1.121	2.702
Recent surgery	0.923	0.972	0.549	1.723
GCS score ≤ 8	0.294	1.623	0.657	4.006

AKI risk was no different in those with positive PE versus those with negative PE (p = 0.386, OR = 0.778, 95% CI = 0.441-1.373). The risk of AKI was also similar in the group who had exposure to intravenous contrast (CTPA) when compared with those who received no intravenous contrast (V/Q scan) (p = 0.542, OR = 0.606, 95% CI = 0.121-3.034) (Table 4). Based on univariate logistic regression, AKI was a predictor of 30-day mortality among the samples (p-value < 0.001, OR = 4.754, 95% CI = 2.67-8.40) (Table 5).

**Table 4:** Diagnostic modalities and the presence of PE in the logistic regression model as predictors of AKI

Variables	P-value	OR	95% CI	
			Lower	Upper
PE	0.386	0.778	0.441	1.373
CTPA	0.542	0.606	0.121	3.034
V/Q scan	0.917	0.921	0.194	4.367

**Table 5:** Acute kidney injury as a predictor of mortality in univariate logistic regression

Variable	P-value	OR	95% CI	
			Lower	Upper
AKI	<0.001	4.745	2.678	8.405

## Discussion

The study aimed to identify the incidence of AKI in patients suspected to have PE, which was found to be 15.8%. Furthermore, 16 (13.4%) of the AKI group were diagnosed with PE. Similarly, a multicenter study conducted with a large sample of confirmed acute PE reported a higher prevalence of AKI (29.5%) with varying degrees of AKI severity [7]. The setting of the patients and the clinical status may contribute to the incidence of AKI. In our study, patients from different hospital settings were included - the emergency department, inpatients, and ICU. It is believed that critically ill patients are more vulnerable to developing AKI. In the literature, the incidence of AKI in the ICU ranged from 3.2% to 67.2% [8]. The high prevalence of AKI in ICU patients is related to multifactorial etiologies; for example, decreased mean arterial pressure, diuretics, and vasopressors were associated with AKI [9]. The contrast-induced nephropathy incidence in ICU patients ranged from 11.5% to 19% [8]. In comparison to the ICU, according to Cho et al. [10], the rate of AKI in patients in the ED with suspected PE and who went through CTPA was 6.49%. In addition to the setting, the criteria used in defining AKI can contribute to different incidence rates [11]. Despite the settings, the criteria, and the presence of PE, the reported rates of AKI in patients with PE or suspected PE were 4.9% and 29.5%, respectively [7,8,10,12-14].

Our findings showed that of the sample (n = 752) with suspected PE, 121 had a confirmed PE diagnosis with a CTPA (90%) and V/Q scan (9.7%). CTPA was the modality of choice in patients with PE suspicion and was chosen for most of the sample (91.3%). The reason for selecting CTPA is its high sensitivity and specificity, with the Prospective Investigation of Pulmonary Embolism Diagnosis II (PIOPED II) trial demonstrating a sensitivity of 83% and a specificity of 96% [15,16]. The V/Q scan was only used for a small proportion (8.7%) and only in situations where the CTPA could not be used, such as in patients with renal insufficiency, contrast allergy, or pregnancy. There was no association between CTPA and AKI (p-value = 0.303). Despite a known association between contrast and nephropathy, there was no statistical significance between the groups who underwent CTPA and those who did not do CTPA, and AKI in the multivariate logistic regression. Our findings were similar to a meta-analysis study conducted by McDonald et al. [17]. Another study noted that using a CT scan to diagnose PE was associated with a low risk of AKI [7]. Different diagnostic modalities with contrast may have a different relation to nephropathy; for instance, IV dye administration in stroke patients was not associated with new or worsening AKI [18]. The discrepancies in the findings related to CI-AKI are possibly associated with the multiple definitions of AKI and poorly designed studies, which could have led to the overestimation of the AKI incidence [8].

We compared the AKI incidence and some of the comorbidities in the current study. There was a statistical significance between DM, HTN, and AKI groups (p-value < 0.001). However, although DM and HTN are known to have a chronic and significant effect on renal function, increasing the vulnerability to AKI [19], they were not predictive of AKI in the multivariate logistic regression. The AKI incidence was higher in patients older than 60 years, which was statistically significant (p-value = 0.0001). This statistical finding can be explained by the age-related loss of kidney function, progressive decrease in the glomerular filtration rate, and renal blood flow [20]. Although the KDIGO AKI guideline concludes that being female is a risk factor for AKI [21], the AKI risk of the female group in the current study decreased by 50% compared with the male group (p-value = 0.004, OR = 0.522, 95% CI = 0.337-0.81). Our finding is similar to studies done in the USA [7,22,23]. It has been suggested that the female gender is reno-protective due to the effects of the sex hormones on the cellular processes instrumental in the pathogenesis of AKI [24]. A history of malignancy was a predictor of AKI and increased the risk (p-value = 0.014, OR = 1.74, 95% CI = 1.21-2.70). We hypothesize the reason might be due to a complication of cancer or the treatment. Patients with cancer are at higher risk of developing infections, sepsis, tumor lysis syndrome, and drug-associated toxicity that significantly increase the risk of developing AKI [25-27]. Other reported factors associated with AKI were heart failure, atrial fibrillation, hematological disorders, and massive/severe PE [7,10,13].

The literature suggested that the hemodynamic changes in patients with PE, such as pulmonary hypertension, increased intra-abdominal pressure, and right ventricular dysfunction, might promote AKI development [28,29]. In a case report, embolectomy reversed the oliguria in a patient with massive PE. This finding supports the hypothesis that PE causes AKI [30]. However, there was no association between AKI and PE in our study. For a long time, AKI was considered a benign condition, which can resolve with supportive care and dialysis. However, recent studies proved that AKI independently increases the risk of mortality and morbidity [7,31,32]. In the current study, 16 (2.1%) patients underwent dialysis within one month of the AKI incidence, and the total mortality rate was 20.2%. AKI was a predictor of mortality within 30 days in our study. It is now accepted that AKI has a substantial negative impact on morbidity and mortality, although the mechanism is unclear. Some studies proposed that AKI leads to acute lung injury (ALI), which may be complicated by cardiogenic/non-cardiogenic pulmonary edema [33]. In addition, respiratory complications requiring mechanical ventilation significantly increase the mortality rate [34-36].

Several limitations have been encountered in this study. We have included patients from different settings, such as inpatient, ED, and ICU, which could contribute to magnifying the incidence rate since critically ill patients are more prone to AKI. The stability of the patients represented by vitals was not included in the study. The type of contrast used, dose, and duration were not assessed.

## Conclusions

In conclusion, the overall incidence rate of AKI in patients with suspected PE was 16.1%. Comorbidities such as diabetes mellitus and hypertension were associated with AKI, but the risk of AKI requiring dialysis was relatively low (2.1%). We have found that malignancy increases AKI risk, whereas the female gender is protective of AKI. The mortality rate in AKI was higher in the AKI group. There was no relationship between the diagnostic modalities, CTPA or V/Q scan, and PE, and AKI, suggesting that the fear of contrast-induced AKI may be overestimated by clinicians. We believe that multiple factors are implicated in the AKI incidence.

## References

[1] Kumar P, Clark M (2012). Kumar and Clark's clinical medicine 8th edition.

[2] Makris K, Spanou L (2016). Acute kidney injury: definition, pathophysiology and clinical phenotypes. Clin Biochem Rev.

[3] Ronco C, Bellomo R, Kellum JA (2019). Acute kidney injury. Lancet.

[4] Al-Homrany M (2003). Epidemiology of acute renal failure in hospitalized patients: experience from southern Saudi Arabia. East Mediterr Health J.

[5] Moore AJ, Wachsmann J, Chamarthy MR, Panjikaran L, Tanabe Y, Rajiah P (2018). Imaging of acute pulmonary embolism: an update. Cardiovasc Diagn Ther.

[6] Khwaja A (2012). KDIGO clinical practice guidelines for acute kidney injury. Nephron Clin Pract.

[7] Murgier M, Bertoletti L, Darmon M (2019). Frequency and prognostic impact of acute kidney injury in patients with acute pulmonary embolism. Data from the RIETE registry. Int J Cardiol.

[8] Case J, Khan S, Khalid R, Khan A (2013). Epidemiology of acute kidney injury in the intensive care unit. Crit Care Res Pract.

[9] Hoste EA, Doom S, De Waele J, Delrue LJ, Defreyne L, Benoit DD, Decruyenaere J (2011). Epidemiology of contrast-associated acute kidney injury in ICU patients: a retrospective cohort analysis. Intensive Care Med.

[10] Cho A, Kim MJ, You JS (2019). Postcontrast acute kidney injury after computed tomography pulmonary angiography for acute pulmonary embolism. J Emerg Med.

[11] Zappitelli M, Parikh CR, Akcan-Arikan A, Washburn KK, Moffett BS, Goldstein SL (2008). Ascertainment and epidemiology of acute kidney injury varies with definition interpretation. Clin J Am Soc Nephrol.

[12] Murgier M, Fouillet L, Ollier E (2020). How many patients recover from acute kidney injury after pulmonary embolism?. Eur Respir J.

[13] Chang CH, Fu CM, Fan PC (2017). Acute kidney injury in patients with pulmonary embolism: a population-based cohort study. Medicine (Baltimore).

[14] Mitchell AM, Jones AE, Tumlin JA, Kline JA (2012). Prospective study of the incidence of contrast-induced nephropathy among patients evaluated for pulmonary embolism by contrast-enhanced computed tomography. Acad Emerg Med.

[15] Gottschalk A, Stein PD, Goodman LR, Sostman HD (2002). Overview of Prospective Investigation of Pulmonary Embolism Diagnosis II. Semin Nucl Med.

[16] Berdahl CT, Vermeulen MJ, Larson DB, Schull MJ (2013). Emergency department computed tomography utilization in the United States and Canada. Ann Emerg Med.

[17] McDonald JS, McDonald RJ, Comin J, Williamson EE, Katzberg RW, Murad MH, Kallmes DF (2013). Frequency of acute kidney injury following intravenous contrast medium administration: a systematic review and meta-analysis. Radiology.

[18] Demel SL, Grossman AW, Khoury JC (2017). Association between acute kidney disease and intravenous dye administration in patients with acute stroke: a population-based study. Stroke.

[19] Finlay S, Bray B, Lewington AJ, Hunter-Rowe CT, Banerjee A, Atkinson JM, Jones MC (2013). Identification of risk factors associated with acute kidney injury in patients admitted to acute medical units. Clin Med (Lond).

[20] Weinstein JR, Anderson S (2010). The aging kidney: physiological changes. Adv Chronic Kidney Dis.

[21] Kellum JA, Lameire N, Aspelin P (2012). Kidney Disease: Improving Global Outcomes (KDIGO) acute kidney injury work group. KDIGO clinical practice guideline for acute kidney injury. Kidney Int Suppl.

[22] Hsu RK, McCulloch CE, Heung M (2016). Exploring potential reasons for the temporal trend in dialysis-requiring AKI in the United States. Clin J Am Soc Nephrol.

[23] Waikar SS, Curhan GC, Ayanian JZ, Chertow GM (2007). Race and mortality after acute renal failure. J Am Soc Nephrol.

[24] Neugarten J, Golestaneh L, Kolhe NV (2018). Sex differences in acute kidney injury requiring dialysis. BMC Nephrol.

[25] Lameire N, Vanholder R, Van Biesen W, Benoit D (2016). Acute kidney injury in critically ill cancer patients: an update. Crit Care.

[26] Cosmai L, Porta C, Foramitti M, Perrone V, Mollica L, Gallieni M, Capasso G (2021). Preventive strategies for acute kidney injury in cancer patients. Clin Kidney J.

[27] Rosner MH, Perazella MA (2017). Acute kidney injury in patients with cancer. N Engl J Med.

[28] Shibagaki Y, Tai C, Nayak A, Wahba I (2006). Intra-abdominal hypertension is an under-appreciated cause of acute renal failure. Nephrol Dial Transplant.

[29] Damman K, Navis G, Smilde TD, Voors AA, van der Bij W, van Veldhuisen DJ, Hillege HL (2007). Decreased cardiac output, venous congestion and the association with renal impairment in patients with cardiac dysfunction. Eur J Heart Fail.

[30] Dadfarmay S, Wahba IM (2011). Acute kidney injury due to pulmonary embolism: the case for 'congestive renal failure'. NDT Plus.

[31] Menzorov MV, Filimonova VV, Erlikh AD, Barbarash OL, Berns SA, Shmidt EA, Duplyakov DV (2021). Renal dysfunction in patients with pulmonary embolism: data from the SIRENA register. Russ J Cardiol.

[32] Xing X, Liu J, Deng Y (2020). Impact of renal function on the prognosis of acute pulmonary embolism patients: a systematic review and meta-analysis. Expert Rev Respir Med.

[33] Faubel S, Edelstein CL (2016). Mechanisms and mediators of lung injury after acute kidney injury. Nat Rev Nephrol.

[34] Waikar SS, Liu KD, Chertow GM (2007). The incidence and prognostic significance of acute kidney injury. Curr Opin Nephrol Hypertens.

[35] Lins RL, Elseviers MM, Daelemans R (2004). Re-evaluation and modification of the Stuivenberg Hospital Acute Renal Failure (SHARF) scoring system for the prognosis of acute renal failure: an independent multicentre, prospective study. Nephrol Dial Transplant.

[36] Park J, Gage BF, Vijayan A (2005). Use of EPO in critically ill patients with acute renal failure requiring renal replacement therapy. Am J Kidney Dis.

